# Nursing student’s satisfaction with two methods of CBL and lecture-based learning

**DOI:** 10.1186/s12909-023-04028-3

**Published:** 2023-01-21

**Authors:** Masoumeh Shohani, Mohammadreza Bastami, Leili Abedi Gheshlaghi, Abbas Nasrollahi

**Affiliations:** 1 Department of Nursing, Faculty of Nursing and Midwifery, Ilam University of Medical Sciences, Ilam, IR Iran; 2 Non-Communicable Diseases Research Center, Bam University of Medical Sciences, Bam, Iran; 3 Reseach Center of Prevention of Psychological Injuries, Ilam University of Medical Sciences, Ilam, Iran

**Keywords:** CBL, Teaching–learning, Lecture-based learning, Nursing students, Satisfaction

## Abstract

**Background:**

Case-based learning (CBL) is an effective teaching–learning strategy that provides a learning environment similar to actual practice. The aim of the present study was to determine the nursing student's satisfaction with two methods of CBL and lecture-based learning.

**Methods:**

This descriptive observational study was conducted in 2019 in School of Nursing and Midwifery in Ilam. All 128 undergraduate nursing students in the 3rd, 4th, and 5th semesters of nursing were enrolled in the study to compare students' satisfaction with CBL and lecture-based learning. Data collection tools included a demographic information questionnaire and a researcher-made questionnaire consisting of 20 questions based on the Likert scale. Data analysis was performed using SPSS Statistics 22.

**Results:**

More than two thirds of the students (81.3%) found the CBL method was better than lectures. 60% of male students and 62% of female students stated that the CBL method increased their self-confidence compared to the traditional lecture-based learning (*p* < 0.03). Students were very satisfied with the CBL method (9.1 ± 1.1 out of 10). There was no significant relationship between gender-related features, and the mean score of students' satisfaction with the CBL method (*p* > 0.05). However, 4th semester students were more satisfied than other students (*P* = 0.02).

**Conclusion:**

The results show that CBL, as a teaching–learning technique in specialized nursing courses, increases students' satisfaction and self-confidence compared to lecture.

## Background

Nowadays, one of the challenges of nursing and midwifery schools around the world is to train students who are able to think critically, high levels of problem-solving skills and provide comprehensive and complete care in different clinical situations [1, 2].

Nurses have found a gap between theory and clinical practice. One of the proposed ways to close the gap between education and clinical practice is to change the traditional educational system to learning based on new methods [3].

Lecture-based learning methods are suitable for achieving the cognitive goals of lower levels of Bloom's Taxonomy and are not suitable for teaching higher levels which include analysis, evaluation and synthesis and require a comprehensive involvement in learning [4].

Case-based learning (CBL) is one of the strategies that enhances active learning in students and prepares them for clinical care. This method was first introduced at Harvard University and this method is used in many medical fields including nursing [5].

In a particular CBL method, a real situation is presented in the form of a scenario that requires problem-solving and decision-making. This method requires 45–90 min and only one case is raised in each session. The teacher presents the scenario and the students listen carefully and take notes, and students are given the opportunity to think about the case. The teacher then begins with an open-ended question: “What is the topic about?” and asks students to participate in discussions and, if necessary, the teacher provides additional information such as diagnostic tests, symptoms, etc. The role of the teacher in this method is initiating, mediating and facilitating the learning process and guides students towards the objectives of the session [6].

According to the results of review articles, CBL is a form of learning that may include a clinical case, a question, or a problem that requires student thinking. In fact, it is a set of learning objectives, previous information and learning resulting from interventions. It can be included in medical curricula and related fields, thus making a connection between theory and clinical practice and deepening learning [7, 8].

In addition to the importance of learning, one of the most important qualitative parameters in higher education is to pay attention to learners' satisfaction.. Furthermore, determining the level of learners' satisfaction has always been an important criterion for measuring the efficiency of educational systems, because student satisfaction is one of the most important factors in the success of educational courses [9].

Choosing the right education method plays an important role in satisfaction and highlights the need for education providers to choose the appropriate teaching method more than ever. [10]. Therefore, the use of novel and alternative educational methods seems necessary.

Although the CBL method is used in various academic fields, there is less scientific basis and evidence for its design, effects, and evaluation in nursing. Considering the experiences of researchers in nursing education and the importance of reducing the distance between theory and clinical practice, the present study was conducted to determine the nursing students’ satisfaction with two methods of CBL and lecture-based learning.

## Methods

### Study design and aim

This descriptive observational study was conducted in 2019 at the School of Nursing and Midwifery in Ilam to determine the effectiveness of CBL in teaching specialized nursing courses on the learning and satisfaction of nursing students.

### Setting and sample

All 128 undergraduate nursing students in the 3rd, 4th, and 5th semesters of nursing who had selected the elderly/adult nursing unit and critical care nursing and were willing to participate in the study were enrolled in the study using a census method.

### Instruments

Data collection tools in this study included a demographic information questionnaire (age, gender, marital status and semester) and a researcher-made 20-item questionnaire to compare the features of the CBL method and lecture-based learning using 5-point Likert scale questions including strongly agree, agree, neither agree nor disagree, disagree and strongly disagree. Finally, a question was included to determine the level of satisfaction with the CBL method on a scale of 0 to 10. The face validity of questionnaires was determined with the help of 20 nursing students. In addition, the content validity ratio (CVR) and content validity index (CVI) of the questionnaires were assessed by 10 faculty members of the nursing faculty. The reliability of the tool was based on Spearman correlation coefficient.

### Participants and comparison of the two methods

In this study, to compare students' satisfaction with CBL and lecture-based learning, students were assigned into different groups based on semester. 3rd, 4th and 5th semester students chose “Adult and Geriatric Nursing” 2 and 3 and “Critical Care Nursing” courses, respectively. Accordingly, based on the experience of researchers as lecturers, two topics were selected for teaching based on CBL and lecture-based learning for each group of students, respectively. Thus, the topics MI and heart failure for 3rd semester students, stroke and multiple sclerosis for 4th semester students, and acute and chronic kidney failure were adopted for 5th semester students. The topics of each group were presented based on CBL and lecture-based learning in one session, respectively, and each topic took about 45 min. Kaddoura method was used for CBL which includes five steps of case presentation, presentation of different questions by the teacher, creating a free and comfortable atmosphere for learners, participation of all learners in discussions and summarization of contents by the teacher [5]. Thus, first the objectives of teaching and then a specific case was presented and the symptoms of the disease and its clinical manifestations were described to the students and the diagnostic tests including graphs and related blood and urine tests were explained and the students were asked to diagnose the disease. All students were asked to take an active part in the discussions. Then, the disease was diagnosed step by step based on the guidance and participation of students, and at each stage, the symptoms and treatment methods of the disease were discussed at the beginning of the session, and these symptoms and care were matched to that specific case. Various questions were constantly asked about the presented case, and the students gradually became familiar with this case of disease. This situation lasted for 45 min and at the end, the final conclusion was made by the lecturer. In the second part of the session, the teacher taught the second topic to the students for 45 min based on lecture-based learning. At the end of the session, students were asked to complete the study questionnaire.

### Ethical considerations

The ethics committee of Ilam University of Medical Sciences (IR.MEDILAM.REC.1398.034) approved the study. This study was carried out in accordance with the declaration of Helsinki. Participants entered the study voluntarily, consciously and with satisfaction after were provided with both verbal and written information about the study. In addition, informed consent was obtained from all subjects. Their information remained confidential.

### Data analysis

After completing the questionnaires, the extracted data were entered into SPSS Statistics 22. The significance level was considered *P* < 0.05. Descriptive statistics were reported as mean (standard deviation) for quantitative variables and as frequency (percentage) for qualitative variables.

The Chi-square test was used to examine the relationship between the features of the CBL method in each of the questions based on gender, and semester. The normality of quantitative variables was evaluated using Kolmogorov–Smirnov test. This test is used to evaluate the null hypothesis according to which a set of data comes from a normal distribution. An independent t-test was used to examine the relationship between the mean score of students' satisfaction and gender based on the CBL method. However, the ANOVA test was used to examine the relationship between satisfaction scores and semester.

## Results

In the present study, 128 nursing students were recruited. The mean (standard deviation) age of students was 23.3 (4.1) years. About 67% (86 students) were male and the rest were female. More than two thirds of the students (113 students, 88%) were single. In the present study, 5th semester students (52 students, 40.1%), 3rd semester students (43 students, 33.9%) and 4th semester students (32 students, 25.2%) had the highest participation, respectively.

Questions with more than 81% agreement were not removed from.

the questionnaire. The CVR and CVI of the scale were 0.80 and 0.81, respectively, which were confirmed because they were > 0.60. Cronbach’s alpha of the questionnaire was 0.9.

From the viewpoint of the students, the most important features of the CBL method were respectively being closer to reality (86%), and better comprehension (82%) compared to the lecture-based learning (Table 1).

**Table 1:** Description of the frequency (percentage) of the features of the CBL method compared to lecture-based learning from the viewpoint of nursing students of Ilam University of Medical Sciences

Questions of satisfaction	Agree and strongly agree	Neither agree nor disagree	Disagree and strongly disagree
1. Covers objectives well	104(81.3)	17(13.3)	7(5.5)
2. It is more interesting	96(75)	23(18)	9(7)
3. Increases students' comprehension	104(81.9)	20(15.7)	3(2.4)
4. More cooperation and participation from students	100(78.1)	25(19.5)	3(2.3)
5. It is closer to reality	109(85.8)	15(11.8)	3(2.4)
6. Increases students' motivation to learn	69(53.9)	34(26.6)	25(19.5)
7. Facilitates students' learning	84(65.6)	37(28.9)	7(5.5)
8. Information is well organized	61(47.7)	43(33.6)	24(18.8)
9. It is more practical	104(81.3)	19(14.8)	5(3.9)
10. The learned material has higher persistence	71(55.9)	34(26.8)	22(17.3)
11. Increases students' visualization power	77(61.1)	37(21.4)	22(17.5)
12. Most nursing courses can be offered through CBL method	86(67.7)	30(23.6)	11(8.7)
13- Increases students' self-confidence	78(60.9)	22(29.7)	21(16.4)
14. Reduces class monotony	104(81.3)	19(14.8)	5(3.9)
15. Makes students think deeply	78(60.9)	27(21.1)	23(18)
16. There is possibility for more questions and answers	81(63.3)	34(26.6)	13(10.2)
17. If this method is used in clinical practice, it will be more efficient	88(68.8)	31(24.2)	9(7)
18. Students' information is well assessed and evaluated	76(59.4)	41(32)	11(8.6)
19. Summarizing the contents is easy	88(68.8)	21(16.4)	19(14.8)
20. In general, CBL method is better than lecture-based learning	104(81.3)	19(14.8)	5(3.9)

There was no significant difference between the features of CBL based on gender in each of the questions except questions 5 and 13 (*p* > 0.05). There was a significant relationship between gender and the feature of being closer to reality (*p* < 0.03); 68 (80%) male students and 41 (97.6%) female students stated that the CBL method has been closer to reality compared to the lecture-based learning. About 60% (52 students) of male students and 62% (26 students) of female students stated that the CBL method increased the students' self-confidence compared to the lecture-based learning (*p* < 0.03).

The distribution of satisfaction scores based on variables of semester and age was normal (*P* > 0.05), so we used ANOVA and independent T-tests, respectively.

Table 2 shows that there was no significant difference between the features of the CBL method in each of the questions based on semester and gender (*p* > 0.05).

**Table 2:** Correlation between student satisfaction score and demographic variables based on CBL method

**Variable**		**Satisfaction M(SD)**^a^	***P*** value**
**Gender**	male	9(1.1)	0.5
	female	9.2(0.9)	
**Educational Semester**	3rd	9.1(1.2)	0.02
	4th	9.4(0.7)	
	5th	8.8(1.1)	

^a^Mean (Standard Deviation)

^**^Using independent t-test and one-way analysis of variance (*ANOVA*)

Overall, more than two thirds of the students (81.3%) found the CBL method better than lecture-based learning. The mean (standard deviation) score of student satisfaction with the CBL method was 9.1 (1.1%), which indicates the high level of student satisfaction with this method. There was no significant relationship between gender and the mean score of student satisfaction with the CBL method (*p* > 0.05). Moreover, 4th semester students were more satisfied with the CBL method compared to other students (*P* = 0.02).

## Discussion

CBL, as a student-centered learning method, provides students with a learning environment similar to actual practice 8).

Several studies have been conducted to compare CBL with the lecture method, which have provided mixed results. Some performed better with CBL [11, 12], some with lecture-based learning [13], and some studies showed no difference between the two methods [14]. The difference in results may be due to the way CBL is performed and the sample size of the studies.

In this study, 81% of the students found the CBL method generally better than the lecture-based learning, which is similar to the findings of other studies. Ma et al. cited CBL as a learning strategy that motivates students to learn, solve problems, and master knowledge in a better way than lecture-based learning [15]. Sangam et al. in an interventional cross-over showed that the mean scores of the CBL group were significantly higher than the lecture-based learning group [16].

In our study, the most important features of the CBL method were being closer to reality, better student comprehension, appropriate coverage of study objectives, applicability of this method and less classroom monotony compared to lecture-based learning. Most previous studies reported that CBL is a powerful tool and educational strategy that improves students', independent learning skills, and ability to prepare for exams, and is a self-directed learning approach [15, 17].

As learning environment plays a crucial role in determining students' satisfaction and readiness to learn, CBL creates a positive learning environment, encourages students to actively discuss and participate in the learning process [18, 19] positive effect of this method in increasing students' self-confidence, which is one of the results of this study and other studies [7, 8].

In addition, the results of this study show that students' satisfaction with this learning method is high. According to other results, this method increases motivation to learn and helps the students to master the necessary knowledge [15] and reduces the monotony of the classroom. Increasing students' attendance and interest in classes is one of the positive effects of this method [20].

The important point is to use this method as an educational strategy in clinical disciplines that enables students to gain clinical competence through case analysis [18, 21], focuses on a well-designed clinical problem through which students identify, research, and link theory and practice to their learning needs [20].

## Conclusion

The results of the study showed that as a teaching–learning method and when compared with lecture-based learning, CBL increases undergraduate student’s satisfaction and self-confidence in specialized courses. CBL can be used as a helpful educational strategy in medical groups. Although a combination of both instructional lectures and case-based learning methods will be effective, more studies are required to assess learning, understanding, and retention of course content to further justify the use of this technique in larger classes and other fields.

## Data Availability

The datasets generated and/or analysed during the current study are not publicly available due to the confidentiality of the participants but are available from the corresponding author on reasonable request.

## Abbreviations

CBL: Case-based learning
MSD: Median standard deviation
ANOVA: Analysis of variance
